# *N*,*N*-Bis(diphenyl­phosphino)ethyl­amine

**DOI:** 10.1107/S1600536809045978

**Published:** 2009-11-14

**Authors:** Nicoline Cloete, Hendrik G. Visser, Andreas Roodt, William F. Gabrielli

**Affiliations:** a Department of Chemistry, University of the Free State, PO Box 339, Bloemfontein 9300, South Africa; b R & D DiVision, Sasol Technology (Pty) Ltd., 1 Klasie Havenga Road, Sasolburg 1947, South Africa

## Abstract

In the title compound, C_26_H_25_NP_2_, the diphenyl­phosphino groups are staggered relative to the PNP backbone, even though the ethyl substituent coordinated to the N atom is not sterically bulky. The N atom adapts an almost planar geometry with two P atoms and a C atom of the allyl group attached to it in order to accommodate the steric bulk of the phenyl groups and the alkyl group. The distortion of the trigonal-pyramidal geometry of the nitro­gen is further illustrated by the bond angles which range between 114.0 (1) and 123.7 (1)°. There are no classical inter­molecular inter­actions.

## Related literature

For similar diphosphineamine non-coordinated ligands with the P—N—P angle ranging between 113.3 (2) and 122.8 (3)°, see: Keat *et al.* (1981 ▶); Cotton *et al.* (1996 ▶); Fei *et al.* (2003 ▶); Cloete *et al.* (2008 ▶).
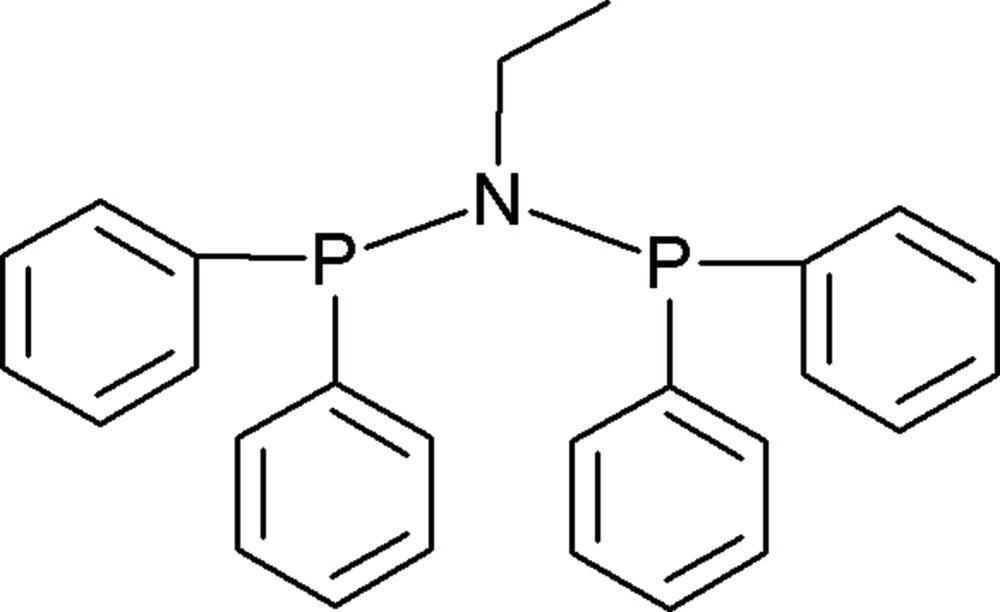


## Experimental

### 

#### Crystal data

C_26_H_25_NP_2_
*M* *_r_* = 413.44
Monoclinic, 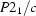

*a* = 9.570 (5) Å
*b* = 13.441 (5) Å
*c* = 16.907 (5) Å
β = 91.647 (5)°
*V* = 2173.9 (15) Å^3^
*Z* = 4
Mo *K*α radiation
μ = 0.21 mm^−1^
*T* = 101 K
0.39 × 0.13 × 0.11 mm

#### Data collection

Bruker X8 APEXII 4K Kappa CCD diffractometer
Absorption correction: multi-scan (*SADABS*; Bruker, 2004 ▶) *T*_min_ = 0.964, *T*_max_ = 0.975
25117 measured reflections
5401 independent reflections
4293 reflections with *I* > 2σ(*I*)
*R*_int_ = 0.046

#### Refinement

*R*[*F* ^2^ > 2σ(*F* ^2^)] = 0.038
*wR*(*F* ^2^) = 0.094
*S* = 1.06
5401 reflections
262 parameters
H-atom parameters constrained
Δρ_max_ = 0.43 e Å^−3^
Δρ_min_ = −0.26 e Å^−3^

### 

Data collection: *APEX2* (Bruker, 2005 ▶); cell refinement: *SAINT-Plus* (Bruker, 2004 ▶); data reduction: *SAINT-Plus* and *XPREP* (Bruker, 2004 ▶); program(s) used to solve structure: *SHELXL97* (Sheldrick, 2008 ▶); program(s) used to refine structure: *SIR97* (Altomare *et al.*, 1999 ▶); molecular graphics: *DIAMOND* (Brandenburg & Putz, 2005 ▶); software used to prepare material for publication: *WinGX* (Farrugia, 1999 ▶).

## Supplementary Material

Crystal structure: contains datablocks global, I. DOI: 10.1107/S1600536809045978/pv2223sup1.cif

Structure factors: contains datablocks I. DOI: 10.1107/S1600536809045978/pv2223Isup2.hkl

Additional supplementary materials:  crystallographic information; 3D view; checkCIF report

## Figures and Tables

**Fig. 1.**
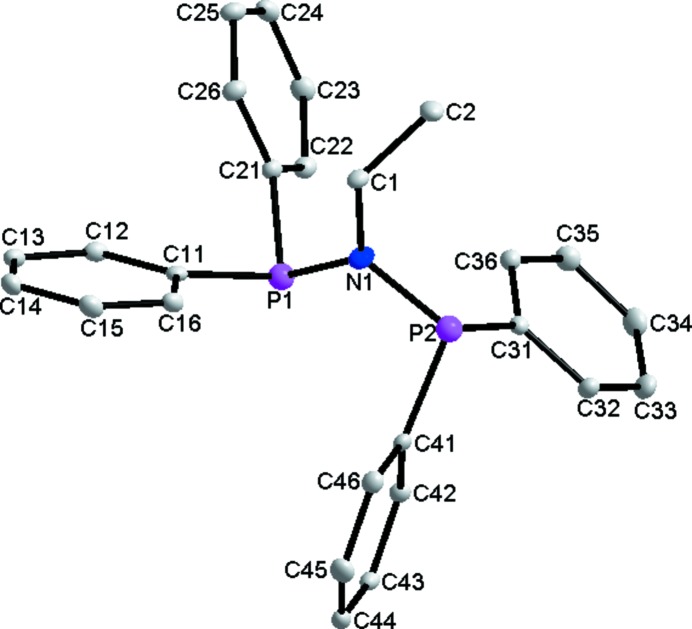
View of (I) (50% probability displacement ellipsoids). H-atoms were omitted for clarity.

**Fig. 2.**
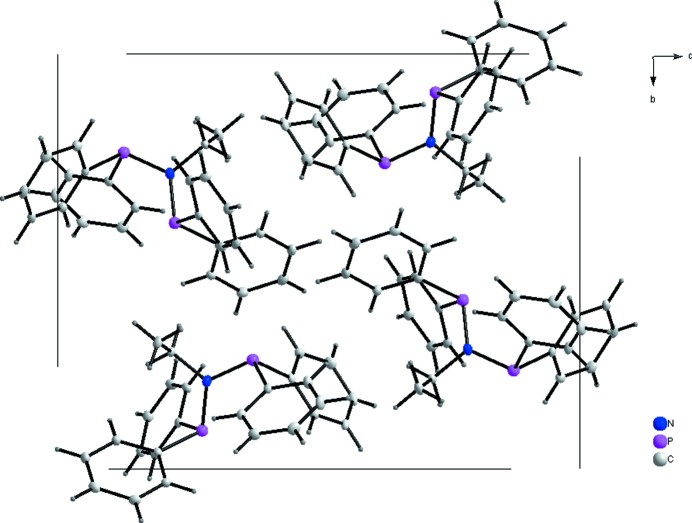
Perspective view of the unit cell of (I) along the a axis.

## supplementary crystallographic information

### Comment

The crystal structure of the title compound, (I), is presented in Figure 1. All bond distances and angles in (I) are normal and fall within the range reported for similar complexes (Keat *et al.*, 1981; Cotton *et al.*, 1996; Fei *et al.*, 2003; Cloete *et al.*, 2008)]. The distance of N1 from the P1—P2—C1 plane is 0.023 (1) Å. The geometry around the phosphorous ligands are distorted from tetrahedral geometry with C—P—C angles being the most distorted (varying from 100.36 (7) to 105.6 (1)°). The P1—N1—P2 angle (123.6 (1)°) is slightly larger than that of other similar compounds quoted above which ranges between 113.3 (2) and 122.8 (3)°. There are no classical intermolecular interactions.

Two conformers are generally found for diphosphineamines and are described (Keat *et al.*, 1981) as C_2v_ and C_s_. In C_2v_ conformer, the phosphorous lone pairs are *cis* with respect to the N—C bond while in the C_s_ conformer the two lone pairs are *trans* relative to the N—C bond. It has been postulated (Keat *et al.*, 1981) that the C_s_ conformer is usually observed for diphoshineamines with relatively bulky substituents on the nitrogen atom. The title compound (I), however has a C_s_ conformer in solid state even though the ethyl group is not particularly bulky.

### Experimental

Ethylpropylamine (0.010 mol, 0.45 g) was dissolved in dichloromethane (30 ml) and placed on an ice bath and triethylamine (0.030 mol, 4.22 ml) was added to the solution while being stirred. Chlorodiphenylphosphine (0.020 mol, 3.62 ml) was slowly added to the reaction mixture. The ice bath was removed after 30 minutes and the reaction mixture was allowed to stir at room temperature for a further 12 h. The dichloromethane was removed under reduced pressure. A mixture of hexane (20 ml) and toluene (2 ml) was added to the remaining white powder and was passed through a column containing neutral activated alumina (35 g). The solvent of the eluent was removed under reduced pressure and the white precipitate was collected. The product was recrystallized from methanol. Single colourless crystals were obtained (yield 2.439 g, 59.0%) the next day which were suitable for X-ray crystallography.

### Refinement

The methylene, methyl and aryl H atoms were placed in geometrically idealized positions with distances C—H = 0.99 0.98 and 0.95 Å, respectively and constrained to ride on their parent atoms, with *U*_iso_(H) = 1.5*U*_eq_(C-methyl) and 1.2*U*_eq_(C-non-methyl).

### Crystal data

**Table:**

C_26_H_25_NP_2_	*F*(000) = 872
*M**_r_* = 413.44	*D*_x_ = 1.263 Mg m^−^^3^
Monoclinic, *P*2_1_/*c*	Mo *K*α radiation, λ = 0.71069 Å
Hall symbol: -P 2ybc	Cell parameters from 5486 reflections
*a* = 9.570 (5) Å	θ = 2.6–27.9°
*b* = 13.441 (5) Å	µ = 0.21 mm^−^^1^
*c* = 16.907 (5) Å	*T* = 101 K
β = 91.647 (5)°	Needle, colourless
*V* = 2173.9 (15) Å^3^	0.39 × 0.13 × 0.11 mm
*Z* = 4	

### Data collection

**Table:**

Bruker X8 APEXII 4K Kappa CCD diffractometer	4293 reflections with *I* > 2σ(*I*)
ω and φ scans	*R*_int_ = 0.046
Absorption correction: multi-scan (*SADABS*; Bruker, 2004)	θ_max_ = 28.3°, θ_min_ = 1.9°
*T*_min_ = 0.964, *T*_max_ = 0.975	*h* = −12→12
25117 measured reflections	*k* = −17→17
5401 independent reflections	*l* = −22→22

### Refinement

**Table:**

Refinement on *F*^2^	0 restraints
Least-squares matrix: full	H-atom parameters constrained
*R*[*F*^2^ > 2σ(*F*^2^)] = 0.038	*w* = 1/[σ^2^(*F*_o_^2^) + (0.0363*P*)^2^ + 0.8849*P*] where *P* = (*F*_o_^2^ + 2*F*_c_^2^)/3
*wR*(*F*^2^) = 0.094	(Δ/σ)_max_ < 0.001
*S* = 1.06	Δρ_max_ = 0.43 e Å^−^^3^
5401 reflections	Δρ_min_ = −0.26 e Å^−^^3^
262 parameters	

### Special details

**Table:**

Geometry. All e.s.d.'s (except the e.s.d. in the dihedral angle between two l.s. planes) are estimated using the full covariance matrix. The cell e.s.d.'s are taken into account individually in the estimation of e.s.d.'s in distances, angles and torsion angles; correlations between e.s.d.'s in cell parameters are only used when they are defined by crystal symmetry. An approximate (isotropic) treatment of cell e.s.d.'s is used for estimating e.s.d.'s involving l.s. planes.

### Fractional atomic coordinates and isotropic or equivalent isotropic displacement parameters (Å^2^)

**Table:**

	*x*	*y*	*z*	*U*_iso_*/*U*_eq_	
N1	0.35096 (13)	0.21232 (9)	0.71479 (7)	0.0155 (3)	
P1	0.41307 (4)	0.09328 (3)	0.72417 (2)	0.01616 (10)	
P2	0.29406 (4)	0.26318 (3)	0.62672 (2)	0.01542 (10)	
C1	0.34840 (16)	0.28240 (11)	0.78268 (8)	0.0177 (3)	
H1A	0.3953	0.3451	0.7678	0.021*	
H1B	0.402	0.2532	0.8279	0.021*	
C2	0.20138 (17)	0.30599 (14)	0.80843 (9)	0.0250 (4)	
H2A	0.2059	0.3523	0.8532	0.037*	
H2B	0.1551	0.2445	0.8245	0.037*	
H2C	0.1483	0.3364	0.7643	0.037*	
C11	0.32088 (16)	0.04094 (11)	0.80923 (9)	0.0174 (3)	
C12	0.22044 (17)	−0.03189 (12)	0.79329 (9)	0.0201 (3)	
H12	0.2041	−0.0532	0.7403	0.024*	
C13	0.14338 (17)	−0.07416 (12)	0.85343 (10)	0.0230 (3)	
H13A	0.0756	−0.1239	0.8413	0.028*	
C14	0.16600 (17)	−0.04328 (12)	0.93092 (9)	0.0234 (4)	
H14	0.1126	−0.0708	0.972	0.028*	
C15	0.26699 (17)	0.02804 (12)	0.94820 (9)	0.0231 (4)	
H15	0.2832	0.0488	1.0014	0.028*	
C16	0.34485 (17)	0.06956 (12)	0.88827 (9)	0.0206 (3)	
H16	0.4147	0.1176	0.901	0.025*	
C21	0.58795 (16)	0.11232 (11)	0.76925 (8)	0.0169 (3)	
C22	0.65088 (16)	0.03565 (12)	0.81394 (9)	0.0193 (3)	
H22	0.5985	−0.0224	0.8252	0.023*	
C23	0.78758 (16)	0.04296 (12)	0.84186 (9)	0.0203 (3)	
H23	0.8288	−0.0104	0.8711	0.024*	
C24	0.86469 (17)	0.12790 (13)	0.82738 (9)	0.0215 (3)	
H24	0.958	0.1337	0.8475	0.026*	
C25	0.80438 (17)	0.20455 (12)	0.78320 (9)	0.0220 (3)	
H25	0.8568	0.263	0.7732	0.026*	
C26	0.66815 (16)	0.19654 (12)	0.75358 (9)	0.0196 (3)	
H26	0.629	0.2488	0.7223	0.024*	
C31	0.13695 (15)	0.19368 (11)	0.59763 (8)	0.0167 (3)	
C32	0.07951 (17)	0.20898 (13)	0.52136 (9)	0.0222 (3)	
H32	0.1242	0.2531	0.4862	0.027*	
C33	−0.04139 (18)	0.16056 (14)	0.49693 (10)	0.0279 (4)	
H33	−0.078	0.1704	0.4447	0.034*	
C34	−0.10978 (18)	0.09766 (13)	0.54801 (10)	0.0277 (4)	
H34	−0.1927	0.0642	0.5309	0.033*	
C35	−0.05620 (17)	0.08398 (13)	0.62401 (10)	0.0248 (4)	
H35	−0.1032	0.0417	0.6595	0.03*	
C36	0.06593 (16)	0.13174 (12)	0.64867 (9)	0.0196 (3)	
H36	0.1015	0.122	0.7011	0.024*	
C41	0.41629 (16)	0.21875 (11)	0.55304 (8)	0.0161 (3)	
C42	0.52940 (16)	0.28007 (12)	0.53709 (9)	0.0205 (3)	
H42	0.5438	0.3394	0.5668	0.025*	
C43	0.62142 (17)	0.25524 (13)	0.47801 (10)	0.0247 (4)	
H43	0.6984	0.2974	0.4676	0.03*	
C44	0.60062 (17)	0.16908 (13)	0.43447 (9)	0.0234 (4)	
H44	0.6631	0.1524	0.3939	0.028*	
C45	0.48928 (17)	0.10702 (12)	0.44978 (9)	0.0201 (3)	
H45	0.4755	0.0477	0.42	0.024*	
C46	0.39761 (16)	0.13181 (12)	0.50904 (9)	0.0184 (3)	
H46	0.3214	0.089	0.5196	0.022*	

### Atomic displacement parameters (Å^2^)

**Table:**

	*U*^11^	*U*^22^	*U*^33^	*U*^12^	*U*^13^	*U*^23^
N1	0.0194 (6)	0.0161 (6)	0.0110 (6)	0.0014 (5)	−0.0010 (5)	−0.0018 (5)
P1	0.0210 (2)	0.01536 (19)	0.01218 (18)	−0.00001 (16)	0.00139 (15)	−0.00045 (14)
P2	0.0176 (2)	0.0164 (2)	0.01224 (18)	0.00027 (15)	0.00057 (15)	−0.00007 (14)
C1	0.0223 (8)	0.0177 (7)	0.0130 (7)	0.0001 (6)	−0.0008 (6)	−0.0030 (6)
C2	0.0246 (9)	0.0316 (9)	0.0187 (8)	0.0050 (7)	0.0009 (7)	−0.0062 (7)
C11	0.0200 (8)	0.0170 (7)	0.0152 (7)	0.0025 (6)	0.0012 (6)	0.0014 (6)
C12	0.0239 (8)	0.0180 (8)	0.0185 (7)	0.0004 (6)	−0.0001 (6)	−0.0010 (6)
C13	0.0209 (8)	0.0202 (8)	0.0280 (8)	−0.0029 (7)	0.0021 (7)	0.0012 (7)
C14	0.0227 (8)	0.0242 (8)	0.0238 (8)	0.0040 (7)	0.0080 (7)	0.0053 (7)
C15	0.0288 (9)	0.0249 (9)	0.0159 (7)	0.0021 (7)	0.0045 (7)	0.0009 (6)
C16	0.0245 (8)	0.0207 (8)	0.0165 (7)	−0.0019 (7)	0.0004 (6)	0.0002 (6)
C21	0.0197 (8)	0.0190 (7)	0.0122 (7)	0.0023 (6)	0.0042 (6)	−0.0009 (6)
C22	0.0236 (8)	0.0170 (8)	0.0176 (7)	0.0008 (6)	0.0046 (6)	0.0004 (6)
C23	0.0226 (8)	0.0223 (8)	0.0161 (7)	0.0062 (7)	0.0047 (6)	0.0030 (6)
C24	0.0166 (8)	0.0264 (8)	0.0217 (8)	0.0028 (7)	0.0031 (6)	0.0004 (7)
C25	0.0203 (8)	0.0205 (8)	0.0254 (8)	−0.0004 (6)	0.0068 (7)	0.0029 (7)
C26	0.0211 (8)	0.0198 (8)	0.0183 (7)	0.0037 (6)	0.0050 (6)	0.0035 (6)
C31	0.0162 (7)	0.0191 (7)	0.0148 (7)	0.0029 (6)	0.0003 (6)	−0.0018 (6)
C32	0.0198 (8)	0.0309 (9)	0.0159 (7)	0.0022 (7)	0.0006 (6)	0.0031 (6)
C33	0.0218 (9)	0.0420 (11)	0.0197 (8)	0.0015 (8)	−0.0055 (7)	−0.0016 (7)
C34	0.0195 (8)	0.0311 (10)	0.0323 (9)	−0.0035 (7)	−0.0039 (7)	−0.0043 (8)
C35	0.0221 (8)	0.0249 (9)	0.0274 (9)	−0.0031 (7)	0.0025 (7)	0.0013 (7)
C36	0.0184 (8)	0.0235 (8)	0.0169 (7)	0.0018 (6)	−0.0005 (6)	−0.0004 (6)
C41	0.0173 (7)	0.0195 (7)	0.0114 (6)	0.0021 (6)	−0.0006 (6)	0.0024 (6)
C42	0.0191 (8)	0.0228 (8)	0.0193 (7)	−0.0019 (6)	−0.0012 (6)	0.0011 (6)
C43	0.0180 (8)	0.0301 (9)	0.0262 (8)	−0.0014 (7)	0.0049 (7)	0.0053 (7)
C44	0.0198 (8)	0.0344 (10)	0.0159 (7)	0.0093 (7)	0.0031 (6)	0.0063 (7)
C45	0.0236 (8)	0.0235 (8)	0.0132 (7)	0.0073 (7)	−0.0007 (6)	−0.0007 (6)
C46	0.0186 (8)	0.0203 (8)	0.0164 (7)	−0.0002 (6)	0.0012 (6)	0.0017 (6)

### Geometric parameters (Å, °)

**Table:**

N1—C1	1.4856 (18)	C23—H23	0.95
N1—P1	1.7127 (14)	C24—C25	1.388 (2)
N1—P2	1.7130 (13)	C24—H24	0.95
P1—C21	1.8369 (18)	C25—C26	1.387 (2)
P1—C11	1.8478 (15)	C25—H25	0.95
P2—C31	1.8255 (17)	C26—H26	0.95
P2—C41	1.8331 (16)	C31—C36	1.391 (2)
C1—C2	1.518 (2)	C31—C32	1.402 (2)
C1—H1A	0.99	C32—C33	1.380 (2)
C1—H1B	0.99	C32—H32	0.95
C2—H2A	0.98	C33—C34	1.386 (2)
C2—H2B	0.98	C33—H33	0.95
C2—H2C	0.98	C34—C35	1.382 (2)
C11—C12	1.393 (2)	C34—H34	0.95
C11—C16	1.403 (2)	C35—C36	1.387 (2)
C12—C13	1.394 (2)	C35—H35	0.95
C12—H12	0.95	C36—H36	0.95
C13—C14	1.385 (2)	C41—C42	1.393 (2)
C13—H13A	0.95	C41—C46	1.394 (2)
C14—C15	1.386 (2)	C42—C43	1.391 (2)
C14—H14	0.95	C42—H42	0.95
C15—C16	1.392 (2)	C43—C44	1.384 (2)
C15—H15	0.95	C43—H43	0.95
C16—H16	0.95	C44—C45	1.383 (2)
C21—C26	1.397 (2)	C44—H44	0.95
C21—C22	1.403 (2)	C45—C46	1.391 (2)
C22—C23	1.382 (2)	C45—H45	0.95
C22—H22	0.95	C46—H46	0.95
C23—C24	1.385 (2)		
			
C1—N1—P1	122.29 (10)	C24—C23—H23	119.9
C1—N1—P2	114.00 (10)	C23—C24—C25	119.44 (16)
P1—N1—P2	123.65 (7)	C23—C24—H24	120.3
N1—P1—C21	102.58 (7)	C25—C24—H24	120.3
N1—P1—C11	104.81 (7)	C26—C25—C24	120.55 (15)
C21—P1—C11	100.36 (7)	C26—C25—H25	119.7
N1—P2—C31	105.59 (7)	C24—C25—H25	119.7
N1—P2—C41	105.52 (7)	C25—C26—C21	120.67 (15)
C31—P2—C41	100.78 (7)	C25—C26—H26	119.7
N1—C1—C2	112.95 (13)	C21—C26—H26	119.7
N1—C1—H1A	109	C36—C31—C32	118.21 (15)
C2—C1—H1A	109	C36—C31—P2	123.55 (12)
N1—C1—H1B	109	C32—C31—P2	118.08 (12)
C2—C1—H1B	109	C33—C32—C31	120.61 (15)
H1A—C1—H1B	107.8	C33—C32—H32	119.7
C1—C2—H2A	109.5	C31—C32—H32	119.7
C1—C2—H2B	109.5	C32—C33—C34	120.51 (16)
H2A—C2—H2B	109.5	C32—C33—H33	119.7
C1—C2—H2C	109.5	C34—C33—H33	119.7
H2A—C2—H2C	109.5	C35—C34—C33	119.42 (16)
H2B—C2—H2C	109.5	C35—C34—H34	120.3
C12—C11—C16	118.04 (14)	C33—C34—H34	120.3
C12—C11—P1	117.34 (11)	C34—C35—C36	120.32 (15)
C16—C11—P1	124.63 (12)	C34—C35—H35	119.8
C11—C12—C13	121.46 (14)	C36—C35—H35	119.8
C11—C12—H12	119.3	C35—C36—C31	120.88 (15)
C13—C12—H12	119.3	C35—C36—H36	119.6
C14—C13—C12	119.74 (15)	C31—C36—H36	119.6
C14—C13—H13A	120.1	C42—C41—C46	118.76 (14)
C12—C13—H13A	120.1	C42—C41—P2	116.96 (12)
C13—C14—C15	119.70 (14)	C46—C41—P2	124.14 (12)
C13—C14—H14	120.2	C43—C42—C41	120.57 (15)
C15—C14—H14	120.2	C43—C42—H42	119.7
C14—C15—C16	120.56 (15)	C41—C42—H42	119.7
C14—C15—H15	119.7	C44—C43—C42	119.90 (15)
C16—C15—H15	119.7	C44—C43—H43	120.1
C15—C16—C11	120.48 (15)	C42—C43—H43	120.1
C15—C16—H16	119.8	C45—C44—C43	120.33 (14)
C11—C16—H16	119.8	C45—C44—H44	119.8
C26—C21—C22	117.87 (15)	C43—C44—H44	119.8
C26—C21—P1	122.22 (12)	C44—C45—C46	119.69 (15)
C22—C21—P1	119.53 (12)	C44—C45—H45	120.2
C23—C22—C21	121.29 (15)	C46—C45—H45	120.2
C23—C22—H22	119.4	C45—C46—C41	120.75 (14)
C21—C22—H22	119.4	C45—C46—H46	119.6
C22—C23—C24	120.15 (15)	C41—C46—H46	119.6
C22—C23—H23	119.9		
			
C1—N1—P1—C21	−53.54 (12)	C22—C23—C24—C25	−1.4 (2)
P2—N1—P1—C21	123.41 (9)	C23—C24—C25—C26	−0.1 (2)
C1—N1—P1—C11	50.91 (13)	C24—C25—C26—C21	1.6 (2)
P2—N1—P1—C11	−132.13 (9)	C22—C21—C26—C25	−1.6 (2)
C1—N1—P2—C31	−116.42 (11)	P1—C21—C26—C25	−174.42 (11)
P1—N1—P2—C31	66.40 (10)	N1—P2—C31—C36	14.23 (15)
C1—N1—P2—C41	137.38 (10)	C41—P2—C31—C36	123.86 (13)
P1—N1—P2—C41	−39.80 (11)	N1—P2—C31—C32	−170.32 (12)
P1—N1—C1—C2	−111.26 (14)	C41—P2—C31—C32	−60.70 (13)
P2—N1—C1—C2	71.52 (15)	C36—C31—C32—C33	−2.5 (2)
N1—P1—C11—C12	109.42 (13)	P2—C31—C32—C33	−178.19 (13)
C21—P1—C11—C12	−144.47 (12)	C31—C32—C33—C34	1.4 (3)
N1—P1—C11—C16	−70.44 (15)	C32—C33—C34—C35	0.3 (3)
C21—P1—C11—C16	35.67 (15)	C33—C34—C35—C36	−0.8 (3)
C16—C11—C12—C13	1.3 (2)	C34—C35—C36—C31	−0.3 (2)
P1—C11—C12—C13	−178.59 (12)	C32—C31—C36—C35	1.9 (2)
C11—C12—C13—C14	0.3 (2)	P2—C31—C36—C35	177.36 (12)
C12—C13—C14—C15	−1.2 (2)	N1—P2—C41—C42	−93.59 (13)
C13—C14—C15—C16	0.6 (2)	C31—P2—C41—C42	156.74 (12)
C14—C15—C16—C11	1.0 (2)	N1—P2—C41—C46	90.85 (14)
C12—C11—C16—C15	−1.9 (2)	C31—P2—C41—C46	−18.82 (14)
P1—C11—C16—C15	177.98 (12)	C46—C41—C42—C43	0.4 (2)
N1—P1—C21—C26	−32.09 (13)	P2—C41—C42—C43	−175.41 (12)
C11—P1—C21—C26	−139.97 (12)	C41—C42—C43—C44	0.1 (2)
N1—P1—C21—C22	155.21 (11)	C42—C43—C44—C45	−0.5 (2)
C11—P1—C21—C22	47.33 (13)	C43—C44—C45—C46	0.3 (2)
C26—C21—C22—C23	0.2 (2)	C44—C45—C46—C41	0.3 (2)
P1—C21—C22—C23	173.18 (11)	C42—C41—C46—C45	−0.6 (2)
C21—C22—C23—C24	1.3 (2)	P2—C41—C46—C45	174.89 (12)

## References

[bb1] Altomare, A., Burla, M. C., Camalli, M., Cascarano, G. L., Giacovazzo, C., Guagliardi, A., Moliterni, A. G. G., Polidori, G. & Spagna, R. (1999). *J. Appl. Cryst.* **32**, 115–119.

[bb2] Brandenburg, K. & Putz, H. (2005). *DIAMOND*. Crystal Impact GbR, Bonn, Germany.

[bb3] Bruker (2004). *SAINT-Plus*, *SADABS* and *XPREP*. Bruker AXS Inc., Madison, Wisconsin, USA.

[bb4] Bruker (2005). *APEX2*. Bruker AXS Inc., Madison, Wisconsin, USA.

[bb5] Cloete, N., Visser, H. G., Roodt, A., Dixon, J. T. & Blann, K. (2008). *Acta Cryst.* E**64**, o480. 10.1107/S1600536808001839 PMC2960458 21201505

[bb6] Cotton, F. A., Kuhn, F. E. & Yokochi, A. (1996). *Inorg, Chim. Acta*, **252**, 251–256.

[bb7] Farrugia, L. J. (1999). *J. Appl. Cryst.* **32**, 837–838.

[bb8] Fei, Z., Scopeleti, R. & Dyson, P. J. (2003). *Dalton Trans.* pp. 2772–2779.

[bb9] Keat, R., Manojlovic-Muir, L., Muir, K. W. & Rycroft, D. S. (1981). *J. Chem Soc. Dalton Trans.* pp. 2192–2198.

[bb10] Sheldrick, G. M. (2008). *Acta Cryst.* A**64**, 112–122. 10.1107/S0108767307043930 18156677

